# Resources of Austrian hospital-based departments of neurology and psychiatry for new amyloid-antibody therapies in early Alzheimer dementia: A survey of the Austrian Alzheimer Society

**DOI:** 10.1007/s00508-025-02608-5

**Published:** 2025-09-05

**Authors:** Gerhard Ransmayr, Edith Eicher, Jürgen Mraczansky, Eirini Braoudaki, Michaela Defrancesco, Walter Struhal, Christian Bancher, Peter Dal-Bianco, Josef Marksteiner, Reinhold Schmidt, Elisabeth Stögmann

**Affiliations:** 1 Medical Faculty, Johannes-Kepler-University, Linz, Austria; 2 European Academy of Neurology, Vienna, Austria; 3 Division of Psychiatry I, Department of Psychiatry, Psychotherapy, Psychosomatics and Medical Psychology, Medical University Innsbruck, Innsbruck, Austria; 4 Department of Neurology, University Hospital Tulln, Tulln, Austria; 5 Landesklinikum Horn-Allentsteig, Horn-Allentsteig, Austria; 6 Dept. of Neurology, Medical University of Vienna, Vienna, Austria; 7 Dept. of Psychiatry and Psychotherapy A, General Hospital, Hall, Austria; 8 Dept. of Neurology, Medical University Graz, Graz, Austria; 9 Department of Neurology, Medical University of Vienna, Vienna, Austria; 10 Schöpfstr. 6, 6020 Innsbruck, Austria

**Keywords:** Alzheimer, Amyloid antibody, Therapy, Hospital, Resources

## Abstract

**Background:**

Disease-modifying therapies with amyloid-antibodies will soon be available for patients with early Alzheimer’s disease, which necessitates diagnostic and therapeutic resources in hospital and outpatient settings.

**Methods:**

The Austrian Alzheimer Society developed an online questionnaire to survey Austrian hospital-based departments of neurology and psychiatry regarding resources for amyloid-antibody therapies.

**Results:**

Between May and October 2023, 30 out of 41 neurology (73%) and 12 out of 33 psychiatry departments (36%) responded. The number of first examinations per year and center ranges between 0 in centers accepting only secondary referrals and 500, median 100.

Of the patients 30% (median; range 0–80%) achieve a mini-mental state examination sum score ≥ 22 constituting an early disease stage. First visits comprise medical history, clinical examination, routine blood sampling and neurocognitive screening in 90–100% of patients, magnetic resonance imaging in 90% (median; range 10–100%), amyloid-PET in 5% (0–70%), cerebrospinal fluid analysis (Aß42, Aß40, tau, phospho-tau) in 25% (0–90%) and Apo‑E testing in 2.5% (0–100%). To assess the amyloid status 18 centers (44%) prefer amyloid-PET to lumbar puncture, 20 (49%) vice versa and 15 centers intend to offer amyloid-antibody therapy.

**Summary:**

Differences between centers and federal states were observed regarding hospital-based resources for amyloid-antibody therapies. A substantial need exists for early dementia diagnosis, medical, neuropsychological, nursing and administrative resources, adequate space, access to amyloid-PET and MRI. All centers highlighted the need for structured patient pathways and multidisciplinary care networks, including neurologists, psychiatrists, radiologists, neuropsychologists operating in clinical practice, rehabilitation and dementia care settings.

**Supplementary Information:**

The online version of this article (10.1007/s00508-025-02608-5) contains supplementary material, which is available to authorized users.

## Introduction

Lecanemab is a humanized monoclonal antibody that binds to soluble amyloid-beta protofibrils, clears cerebral amyloid and slows down the decline of neurocognitive functions and activities of daily living in persons with mild cognitive impairment (MCI) and mild dementia due to Alzheimer’s disease (AD) [1]. Marketing is expected this autumn [2].

Donanemab, an immunoglobulin G1 monoclonal antibody directed against insoluble, modified, N‑terminal truncated beta-amyloid, clears cerebral beta-amyloid from amyloid plaques and decelerates the progression of cognitive and functional decline in early AD [3]. The European Medicines Agency has recently recommended the marketing of donanemab in the European Union [4].

Both substances are administered intravenously, lecanemab, at 2‑week and donanemab, at 4‑week intervals. Regular follow-up visits are needed because of the risk of amyloid-related cerebral imaging abnormalities (ARIAs) causing neurological deficits, confusion and seizures, and infusion-related reactions were reported [1, 3].

Based on the current data the risk of ARIAs under amyloid-antibody therapy (AAT) is significantly elevated in persons with Apo‑E Ɛ 4 homozygosity, vascular encephalopathy, cerebral microbleeds and superficial hemosiderosis. These diagnoses, severe autoimmune disorders, clotting disorders, anticoagulation, seizures and also severe psychiatric conditions exclude patients from therapy.

The diagnostic process before AAT comprises medical history, clinical examination, neuropsychological testing, routine laboratory, Apo‑E status, magnetic resonance imaging (MRI) including iron-sensitive sequences (T2* or SWI), amyloid positron emission tomography (A-PET) and/or lumbar puncture to examine the cerebrospinal fluid (CSF) for amyloid-beta (Aß)1-42, Aß1-40, tau and phospho-tau. A check list of exclusion criteria as well as thorough patient information and informed consent of the patient must be completed [1, 5]. Repeated clinical follow-up visits including a minimum of 3 MRI examinations are required during the first 6 months of treatment.

In 2000, the estimated number of individuals diagnosed with dementia in Austria was 90,000 [6] and 19 years later 150,000–160,000 [7]. Alzheimer’s disease dementia (ADD) and related disorders (LATE, PART) [8, 9] contribute to 60–70% of all dementia patients [10]. In around 50% of ADD cases, around 50,000 persons in Austria, dementia is mild [11]. Aβ-positive prodromal AD has recently been estimated to be as high as 193,500 in Austria [12, 13]. Prodromal AD is characterized by MCI including episodic memory impairment (amnestic subtype of MCI, aMCI) not interfering with activities of daily living and converts to ADD in 10–15% per year [12, 13].

Only 5–20% of AD patients might be eligible for AAT [1, 5, 13–19], which is indicated in amyloid-positive aMCI and mild ADD after exclusion of contraindications. The disease-modifying effects are clinically meaningful (significant delay in cognitive decline and impaired activities of daily living after 18 months of treatment); however, beneficial effects are uncertain beyond 18 months of treatment and side effects lead to discontinuation of therapy (in around 15–30% of patients) [1, 3]. Patient inclusion and follow-up procedures are complex and require large resources of medical staff, diagnostic equipment as well as time and space for examinations and the application of AAT infusions. Diagnostic work-up and follow-up need highly qualified centers cooperating with partners in the outpatient setting [5].

The goal of the present study was to evaluate diagnostic and therapeutic resources of hospital-based departments of neurology (DN) and psychiatry (DP) in Austria for AAT.

## Methods

An internet-based survey was designed by the managing board of the Austrian Alzheimer Society (ÖAG) and programmed and maintained by E.B. in 2023 to assess the resources of hospital-based DN and DP for patients with neurocognitive disorders (university, province and district hospitals, convent hospitals with public patient care mandate), as well as at the Department of Geriatric Medicine and Rehabilitation of the Private Medical University of Salzburg (allocated to the DN group because of its neurological focus in patient care) and the Psychosocial Services (*Psychosoziale Dienste*), a multi-center psychiatric out-patient service of the City of Vienna (allocated to the DP group).

Regional representatives of the ÖAG informed individually each head of the DN and DP of the aims of the survey. Detailed information was sent along with the link to the questionnaire. Participants were informed to receive an anonymized statistical summary of the results and gave their informed consent.

The questionnaire comprised the following topics/issues:

- Type of medical service provided and/or planned to be provided.
- Patient frequencies (first examination, follow-up visits) and waiting period until first visit.
- Percentage of persons with mini mental state examination (MMSE) sum score ≥ 22.
- Percentages of patients undergoing the following examinations:
- Medical history, clinical status, routine blood examination, neurocognitive screening, detailed neuropsychological testing, cerebral computed tomography (CCT), MRI, CSF examinations for Aß40, Aß42, tau, phospho-tau, A‑PET and fluorodeoxyglucose PET (FDG-PET), electroencephalography (EEG) and Apo‑E testing.
- Resources for diagnosis and AAT (administrative, medical and neuropsychological staff, nurses, CSF examinations, MRI, A‑PET, room for examinations and infusion therapy).
- Cooperations for patient care including general practitioners, radiologists, neurologists and psychiatrists in practice, medical laboratories, neuropsychologists, occupational and speech therapists, and social services.

We did not assess risk factors of dementia, such as positive family history, vascular risk parameters, lifestyle, alcohol abuse and heart diseases. The survey was carried out from May to October 2023. Incoming data were pseudo-anonymized and, after plausibility checks, analyzed by students of the Medical Faculty, Johannes Kepler University Linz (E.E., J.M.). Descriptive statistics and Spearman rank correlations were used.

## Results

### Participation in the survey

Out of 41 DN 30 (73%) and 12 out of 33 DP (36%) replied to the survey (total response rate 57%). Centers from 8 out of 9 Austrian federal states (8.7 of 9.1 million inhabitants) [20] responded to the survey. Departments from all Austrian university hospitals and more than 50% of central, federal state, district and convent hospitals with public patient care mandate responded, two centers reported precise statistics, the other responding centers estimates.

### Specialized neurocognitive services

Out of 30 DN 29 responded, 18 DN provide both inpatient and outpatient services, 1 solely an outpatient, 4 solely inpatient services, 6 no special neurocognitive service. Four DP provide special inpatient and outpatient services, 5 DP solely an outpatient, 2 solely inpatient services. One DP provides no special neurocognitive service. Seven DN plan to enlarge their resources for patients with neurocognitive disorders, 2 a neurocognitive day-care center. An inpatient ward for dementia patients, a daycare center and a memory out-patient clinic is envisaged by 1 DP each.

### Patient frequencies, referrals, waiting periods and MMSE at first visit (year 2022)

The number of primary referrals of patients complaining of cognitive deficits (response rate 26 out of 30 DN, 9 out of 12 DP) and the percentage of persons with a MMSE sum score of ≥ 22 at the first visit (lower cut-off for inclusion in therapy with lecanemab; response rate 26 out of 30 DN and 11 out of 12 DP) are shown in Table 1. The reported percentages of patients with a MMSE sum score ≥ 22 at the first visit suggest in turn that 20–100% of patients seen at DN and 50–95% of patients at DP suffer from moderate to severe neurocognitive impairment (MMSE < 22) and are not eligible for AAT.

**Table 1:** Percentages and frequencies of visits in the year 2022 and waiting time until 1st visit

	All departments	Departments of neurology	Departments of psychiatry
Number of 1st examinations 2022 (median, range)	100 (0–500)	100 (0–313)	100 (0–580)
MMSE ≥ 22 at first visit (%, median, range)	30 (0–80)	45 (0–80)	20 (5–50)
Waiting time for 1st visit (weeks; median, range)	5 (0–25)	5.0 (0–25)	4.5 (0–20)
Number of follow-up visits per year (median, range)	55 (0–800)	65 (0–508)	30 (0–800)
Follow-up visit on site after 1st visit (%, median, range)	10 (0–95)	17.5 (0–90)	10 (0–95)

*MMSE* mini-mental state examination sum score (maximum 30)

*5 out of 30 participating departments of neurology and 2 out of 12 participating departments of psychiatry did not respond to these questions.

The mean waiting period (in weeks) between booking date and first visit (response rate 24 out of 30 DN, 10 out of 12 DP), the overall number of follow-up visits and actual percentages of persons seen for follow-up visits after a first visit (response rate 26 out of 30 DN and 11 out of 12 DP) are shown in Table 1. The proportion of patients seen after a first visit is variable (DN 0–90, median 17.5%, DP 0–95, median 10%, Table 1). Of DN 32% and 50% DP patients have follow-up visits both on-site and in practices of neurologists, psychiatrists or general practitioners. The remainder of patients have follow-up visits exclusively in practices of neurologists, psychiatrists or general practitioners. Self-referrals are accepted by 30% of DN and 81% of DP.

### Diagnostic modalities, frequencies, diagnostic and therapeutic resources

The percentage of examinations performed at the first visit are shown in Table 2. Only single responses were missing (details not shown), one DN and 4 DP presumably refer patients for CCT and/or MRI to radiologists in practice or do not consider neuroimaging because of pre-existing neuroimaging or other reasons.

**Table 2:** Percentages of patients examined at the 1st visit

	All departments	Departments of neurology	Departments of psychiatry
Median (range)			
1. History taking	100 (100–100)	100 (100–100)	100 (100–100)
2. Clinical status	100 (10–100)	100 (10–100)	90 (50–100)
3. Routine blood and serum parameters, TSH, vitamin B12, folate	100 (0–100)	100 (0–100)	90 (95–100)
4. Cognitive screening	100 (30–100)	100 (30–100)	90 (30–100)
5. Detailed cognitive testing	70 (10–100)	70 (10–100)	50 (15–100)
6. MRI	90 (10–100)	90 (50–100)	65 (10–100)
7. CCT	27.5 (0–100)	27.5 (0–100)	20 (0–100)
8. A‑PET	5 (0–70)	5 (0–70)	5 (0–30)
9. FDG-PET	10 (0–80)	15 (0–80)	5 (0–30)
10. CSF dementia parameters	25 (0–90)	35 (0–90)	7.5 (0–30)
11. Genetic analysis (Apo E)	2.5 (0–100)	5 (0–100)	0 (0–95)
12. EEG	20 (5–100)	30 (5–100)	10 (0–80)

*CSF* cerebrospinal fluid examination (lumbar puncture), *CCT* cerebral computed tomography, *A‑PET* amyloid PET, *EEG* electroencephalography, *FDG-PET* fluorodeoxyglucose-PET, *MRI* magnetic resonance imaging, *PET* positron emission tomography, *TSH* thyroid-stimulating hormone

**Table 3:** Numbers of centers reporting lack of resources for AA therapy

	All departments	Departments of neurology	Departments of psychiatry
Administrative staff	13	12	1
Medical doctors	28	18	10
Nurses	19	13	6
(Neuro)-psychologists	16	14	2
Space (examinations, infusions)	21	16	5
MRI	15	9	6
Amyloid PET	18	11	7

*AA* amyloid antibody, *MRI* magnetic resonance imaging, *PET* positron emission tomography

In 17 DN and in 5 DP, both CCT and MRI are performed. The percentage of patients with MRI correlates negatively with the percentage of individuals with CCT (Spearman rank correlation; rs = −0.3860, two-tailed *p* = 0.0116).

The frequency of advanced AD diagnosis including amyloid status (A-PET and/or CSF for amyloid status), FDG-PET, Apo‑E status and EEG is variable and on average low.

The number of primary referrals in 2022, a parameter of center size, does not correlate with the percentage of patients receiving MRI, A‑PET, Apo‑E testing and lumbar puncture for neurodegenerative CSF parameters (Spearman rank correlation, *p* > 0.05) but with the percentage of individuals getting a FDG-PET (rs = 0.3593, *p* = 0.0340). The percentage of patients receiving an A‑PET did not differ in academic compared to non-academic affiliations; relatively high (≥ 20) percentages of patients with an A‑PET were reported by 2 out of 7 academic and 7 out of 35 non-academic departments.

The MRI baseline and follow-up examinations for patients receiving AAT are considered realizable by 16 out of 30 DN and 7 out of 11 DP, mainly in cooperation with radiologists in practice.

Out of 27 DN 17 prefer CSF examination to A‑PET and 10 DN, vice versa, to assess the amyloid status, 3 out of 11 DP prefer CSF examination rather than A‑PET, 8 vice versa. In summary, 49% of centers responding to this question prefer CSF and 44% A‑PET. In 7 DP lumbar puncture is not routinely performed. If lumbar puncture is indicated, 11 out of 12 DP and 8 out of 22 DN refer patients to inpatient services, 27 out of 30 DN and 11 out of 12 DP responded to the questions about the need for additional diagnostic and therapeutic resources. Most centers reported lack of resources in various fields (see Table 3).

### Cooperations and networks

The DN and DP were asked for existing cooperations and participation in networks with neurologists, psychiatrists, geriatricians, radiologists, psychologists, general practitioners, medical laboratories, psychosocial care units or physical, occupational and speech therapy and 2–9 out of 25 DN and 0–6 out of 11 DP cooperate with 1 or more of these disciplines. All DN and DP underscored the need for multidisciplinary networks for dementia care in general and AAT.

### Correlations between center size and diagnostic capacities, feasibility of AATs

The number of first visits per year correlates with the number of follow-up visits on-site and the proportion of patients with mild AD (MMSE sum scores ≥ 22; Table 4). In 13 DN and 2 DP AAT was considered feasible at their centers, 11 out of these centers in cooperation with medical partners in practice. Small, medium and large centers reported concerns about having sufficient resources for amyloid-antibody therapy (free text responses).

**Table 4:** Stratification of all neurological and psychiatric services in three groups according to frequencies of first visits per year (150–500, 80–120, 0–60 persons)

*First examinations/year*			
*Range*	*150–500*	*80–120*	*0–60*
Median	200	100	42.5
*Follow-up visits on site/year*			
Median	245	50	7.5
Range	0–800	0–200	0–100
*% of 1st visits MMSE ≥* *22*			
Median	40	37.5	20
Range	20–80	10–75	0–75

*MMSE* mini-mental state examination sum score

### Participation in a nationwide Quality of Dementia Care Registry

Out of 38 centers 24 expressed their interest to participate in such a registry planned by the Austrian Ministry for Labor, Social Affairs, Health, Care and Consumer Protection and 20 centers reported concerns regarding the expected workload (free text responses).

### Estimated regional availability of AAT

The survey suggests that DN and DP could care for altogether around 4800 new patients per year. This figure results from the sum of the number of first examinations reported by each DN and DP and corresponds to around 14% of Austrians who, according to the literature, are estimated to convert to dementia per year (35,000) [13, 20, 21]. Not included in this calculation are patient frequencies from hospital-based centers not responding to the survey. These centers, however, might not dispose of major resources and therefore not contribute to a significantly higher percentage of patients cared for. We also did not assess the annual capacities of cooperating neurologists and psychiatrists in practice. The core activities of diagnosis and AAT, however, will most likely be performed in hospital-based centers.

The sum of the reported frequencies of first visits (year 2022) in hospital-based DN and DP from each Austrian federal state (8 out of 9 Austrian federal states) was divided by the population of the respective federal state in thousands (range of inhabitants per federal state 302–2028 × 1000) [20]. The resulting quotient, an approximative measure of the relative availability of hospital-based services for patients with neurocognitive disorders per Austrian federal state, ranges from 0.17 to 0.92, median 0.4. These estimations reveal major differences between Austrian federal states concerning resources for hospital-based care for patients with neurocognitive disorders.

## Discussion

The use of AAT is soon to become available in Austria for patients with early AD. The implementation requires adequate diagnostic and therapeutic infrastructures in hospital-based settings, in cooperation with outpatient services. To assess current preparedness of DN and DP, the Austrian Alzheimer Society conducted this online survey. Even though 43% of the contacted centers did not respond to the survey and most centers reported estimations our study provides an approximative evaluation of present diagnostic and therapeutic activities, resources and needs of DN and DP; however, the spectrum of patients seen in hospital-based dementia care units might differ from that seen in practices of neurologists and psychiatrists.

Both the resources and the activities for dementia care and AAT differ from center to center and between Austrian federal states. They are altogether limited (Table 3) and resources of cooperating neurologists and psychiatrists in practice are unknown. Nevertheless, 15 DN and DP in 7 of 9 federal states consider AATs feasible in their centers.

A median of 30% of patients examined for the first time achieve a MMSE sum score of ≥ 22 (range 0–80%) Conversely, a median of 70% (range 20–100%) of patients are examined for the first time at an advanced stage of neurocognitive decline and are therefore not eligible for AAT. Better awareness of the demand for an early diagnosis of neurocognitive impairment is needed both in the medical field and in the public. Several centers expressed also the need for improved political awareness of dementia care in Austria.

The waiting times were variable and ranged from immediate access to first visit to 6 months (Table 1). Since 2023, however, individual centers reported a dramatic increase in persons asking for short-term diagnostic work-up.

Larger centers have higher proportions of patients with MCI or mild dementia (MMSE ≥ 22), but not necessarily sufficient resources for diagnosis and AAT. Moreover, resources for follow-up visits during AAT are insufficient in most centers.

Relatively high proportions of patients are examined with CCT before MRI or only with CCT. A negative correlation was found between the percentages of patients with MRI and CCT. These findings may suggest variable availability of MRI but CT might also be preferred to MRI in multimorbid and cognitively severely affected subjects or as a primary diagnostic step before MRI to exclude cerebrovascular and space-occupying lesions.

Advanced diagnostic testing including A‑PET or FDG-PET, CSF analyses, EEG and examination of the Apo E status is performed in variable, altogether small proportions of patients (Table 2). In several centers lumbar puncture is rarely practiced. Major proportions of patients are hospitalized for lumbar puncture although patients could be dismissed after few hours of observation [22].

In most centers access to A‑PET is limited and A‑PET is expensive but preferred to lumbar puncture by most centers. Centers without nuclear medicine need to send their patients to other hospitals, which may cause additional costs. The FDG-PET is more frequently performed than amyloid-PET, mainly at larger centers, probably due to better availability and EEG is variably performed, presumably to exclude prion disease or epileptic activity.

Limited resources for AAT are also reported from other countries [23, 24]. In Canada, for instance, referrals to hospital-based DP and DN are reserved for complex cases [23]; special recommendations and programs to support outpatient physicians in referral practice are implemented. An increase of hospital-based resources including improved access to diagnostic modalities, in particular MRI and A‑PET, is urgently needed.

Hospital-based resources vary markedly between Austrian federal states. Around 4800 new patients per year were reported by 57% of all hospital-based DN and DP, which corresponds to 14% of Austrians developing dementia per year (35,000) and 21% converting to ADD [13, 20, 21]. A nationwide plan for improvement of resources is needed. Representatives of the Ministry of Health and Care, public health insurances, local health authorities, the Austrian Alzheimer Society, Neurological Society and Medical Association meet to improve dementia care resources and to prepare the implementation of the new AAT.

## Supplementary Information

Experts and centers participating at the survey
