# Supplementary material for: Resources of Austrian hospital-based departments of neurology and psychiatry for new amyloid-antibody therapies in early Alzheimer dementia: A survey of the Austrian Alzheimer Society
Source: Wien Klin Wochenschr. 2025 Sep 5;138(5-6):137–43. doi: 10.1007/s00508-025-02608-5 (PMC12992369; doi:10.1007/s00508-025-02608-5)
Supplement: Supplementary file 1 — Experts and centers participating at the survey [file 508_2025_2608_MOESM1_ESM.docx]

**Experts and centers participating at the survey:**

Asenbaum-Nan Susanne, Dept. of Neurology, Landesklinikum Amstetten, Amstetten

Barth Martin, Dept. of Psychiatry, Pyhrn-Eisenwurzen Klinikum, Steyr

Berger Otto, Dept. of Neurology, Klinik Favoriten, Vienna

Diez Josef, Dept. of Neurology, Elisabethinen Hospital, Graz

Djamshidian Atbin, Dept. of Neurology, Medical University of Innsbruck, Innsbruck

Erfurt Andreas, Dept. of Psychiatry I, Klinik Hietzing, Vienna

Feichtinger Michael, Dept. of Neurology, Landeskrankenhaus Hochsteiermark, Bruck an der Mur

Ferrari Julia, Dept. of Neurology, Barmherzige Brüder Konvent Hospital, Vienna

Fertl Elisabeth, Dept. of Neurology, Klinik Landstrasse, Vienna

Glück Katharina, Dept. of Psychiatry, Klinikum Wels-Grieskirchen, Wels-Grieskirchen

Guger Michael, Dept. of Neurology, Pyhrn-Eisenwurzen Hospital, Steyr

Hlade Peter, Dept. of Psychiatry, Elisabethinen Hospital, Graz

Höger Franz Stefan, Dept. of Neurology, Province Hospital, LKH II Sigmund Freud, Graz

Iglseder Bernhard, Dept. of Geriatric Medicine and Rehabilitation, Private Medical University, Salzburg

Jagsch Christian, Dept. of Gerontopsychiatry and Geriatric Psychotherapy, Landeskrankenhaus II Sigmund Freud, Graz

Kapeller Peter, Dept. of Neurology, Landeskrankenhaus Villach, Villach

Katzenschlager Regina, Dept. of Neurology and Acute Geriatric Medicine, Klinik Donaustadt, Vienna

Lackner Peter, Dept. of Neurology, Klinik Floridsdorf, Vienna

Lampl Christian, Dept. of Neurology, Barmherzige Brüder Konvent Hospital, Linz

Lugmayer, Dept. of Psychiatry, Salzkammergut Klinikum, Vöcklabruck

Oberndorfer Stefan, Dept. of Neurology, University Hospital, St. Pölten

Pirker Walter, Dept. of Neurology, Klinik Ottakring, Vienna

Pirker-Kees Agnes, Dept. of Neurology, Klinik Hietzing, Vienna

Rujescu Dan, Dept. of Psychiatry, Medical University of Vienna, Vienna

Schlüter Martina, Dept. of Psychiatry, Landesklinik Waidhofen/Thaya, Waidhofen/Thaya

Schnider Peter, Dept. of Neurology, University Hospital, Wiener Neustadt

Seifert-Held Thomas, Dept. of Neurology, LKH Murtal, Knittelfeld-Judenburg, Knittelfeld

Sellner Johann, Dept. of Neurology, Landesklinikum Mistelbach-Gänserndorf, Mistelbach

Sorre Karin, Dept. of Neurology, Klinikum Klagenfurt, Klagenfurt

Staffen Wolfgang, Dept. of Neurology, Private Medical University, Salzburg

Staykov Dimitre, Dept. of Neurology, Barmherzige Brüder Konvent Hospital, Eisenstadt

Sepandj Asita, Psychosoziale Dienste, Vienna

Till Verena, Dept. of Neurology, Salzkammergut Klinikum, Vöcklabruck

Topakian Raffi, Dept of Neurology, Klinikum Wels-Grieskirchen, Wels-Grieskirchen

Vetchy Michael, Dept. of Gerontopsychiatry, Klinik Hietzing, Vienna

Wally Beate, Dept. of Psychiatry and Psychotherapeutic Medicine, Klinik Donaustadt, Vienna

Wührer Susanne, Dept. of Neurology, Elisabethinen Hospital, Graz

Zeller Matthias, Dept. of Neurology, Kepler University Hospital, Linz
